# Slow Down of the Gulf Stream during 1993–2016

**DOI:** 10.1038/s41598-019-42820-8

**Published:** 2019-04-30

**Authors:** Shenfu Dong, Molly O. Baringer, Gustavo J. Goni

**Affiliations:** Physical Oceanography Division, AOML/NOAA, Miami, Florida USA

**Keywords:** Physical oceanography, Physical oceanography

## Abstract

The Gulf Stream, the main heat-carrier from low to high latitudes in the North Atlantic Ocean, influences the climate and weather in the northern hemisphere. In this study we determine and analyze the position, speed, and width of the Gulf Stream (GS) from 80°W–50°W using satellite altimeter sea surface height (SSH) measurements to examine the possible link between changes in the strength of the GS and coastal sea levels along the U.S. East Coast. During our 24-year study period (1993–2016), the GS experienced a southward shift east of 65°W after passing the New England Seamount chain. This southward shift was accompanied by a weakening of the GS, associated with an increase in SSH to the north of the GS. West of 70°W, however, we found no statistically significant trends in the GS properties, consistent with results based on *in situ* measurements. This lack of a trend to the west fails to support a direct link between a long-term slowdown of the GS west of 70°W and sea level rise acceleration along the U.S. East Coast, though a slowdown of the GS east of 65°W may contribute to sea level rise. It is also possible that heat carried to the region by the GS may be responsible for these observed sea level changes.

## Introduction

The Gulf Stream (GS), the western boundary current in the North Atlantic, influences the weather and climate patterns of eastern North America and Western Europe through both hydrodynamic and thermodynamic processes^1–3^. As the northward flowing upper limb of the Atlantic Meridional Overturning Circulation (AMOC), the GS transports a large amount of heat from the low to high latitudes (~2.5 PW at 26.5°N across Florida Straits^4^, 1 PW = 10^15^ W), most of which is released to the atmosphere in the midlatitudes^5^. Changes in the GS system have been linked to changes in various weather and climate phenomena such as storm tracks over the northwest Atlantic Ocean, the AMOC, and the weather in Europe^2,3,6–10^. Although questions remain as to whether the acceleration in sea level rise along the U.S. East Coast between Cape Hatteras and Cape Cod, as observed by tide gauges, reflects a long-term trend or multi-decadal ocean dynamics variability^11,12^, recent studies have linked this sea level rise acceleration to a slowdown of the GS through dynamic response^13–16^. For example, using a sea surface height (SSH) gradient as an indication of GS strength, Ezer *et al*.^15^ argued that weakening of the GS during 1993–2011, as observed by satellite altimetry in the region 75.5°W–70°W, corresponded to a reduced SSH difference across the GS. This reduced SSH difference resulted in an increase in SSH to the north of the GS and a decrease in SSH to the south. However, Rossby *et al*.^17^ found no significant decrease in GS strength over a similar time period (1993–2012) based on *in situ* subsurface velocity measurements at approximately 70°W.

**Figure 1:**
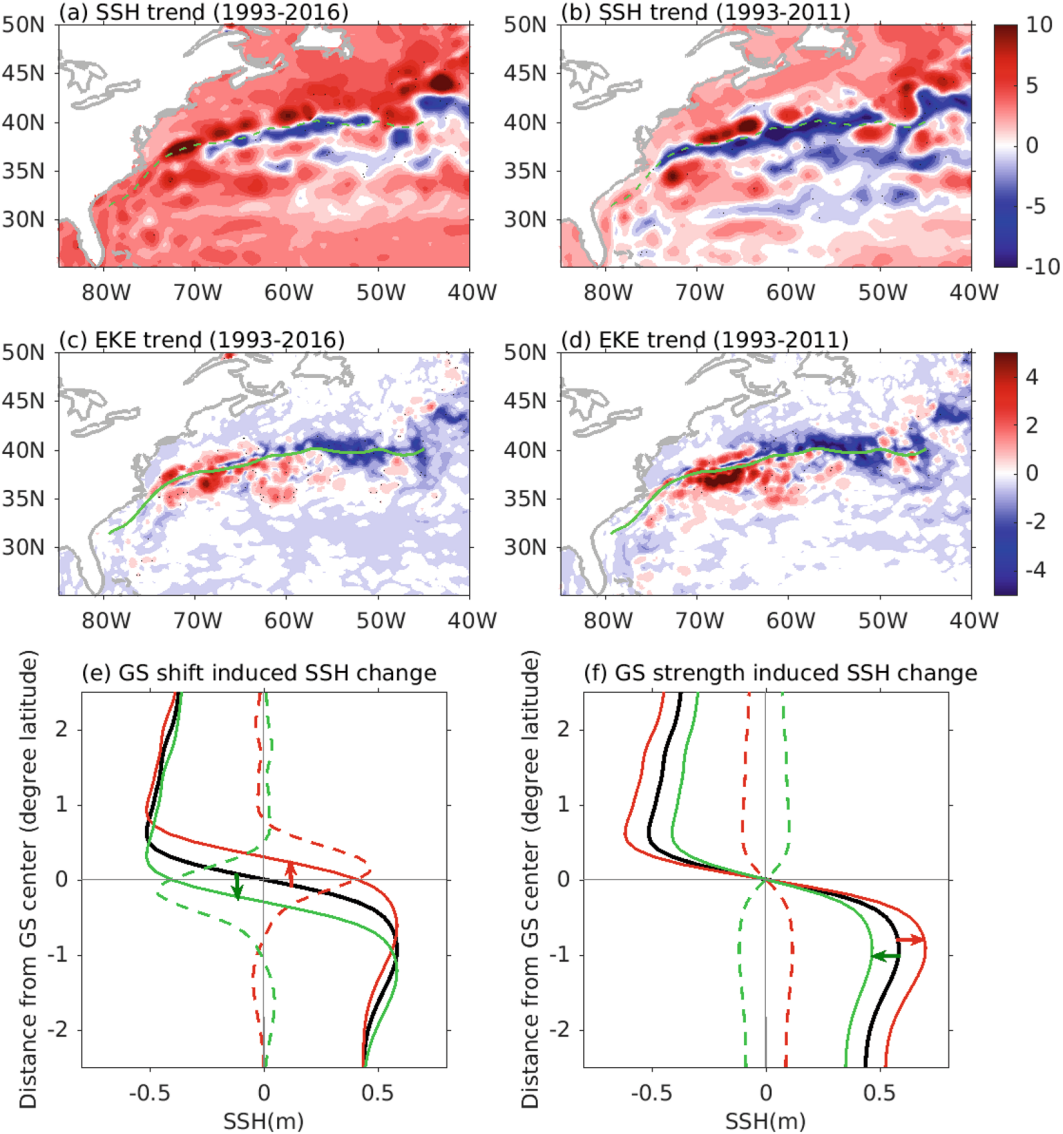
Linear trends in SSH during (**a**) 1993–2016 and (**b**) 1993–2011, and the trends in EKE during (**c**) 1993–2016 and (**d**) 1993–2011. Green curves in (**a**–**d**) denote the mean GS position. Low panels (e,f) demonstrate the SSH changes induced by meridional shifts in the GS position and changes in the GS strength. Black lines indicate the long-term mean, red lines denote the northward shift (**e**) or strengthening (**f**), and green lines correspond to a southward shift (**e**) or weakening (**f**). Units are cm/decade for SSH trend and cm^2^/s^2^/decade for EKE trend.

A number of factors can contribute to these discrepancies in GS strength, including the use of data from different observing platforms, different definitions for GS strength, and spatial variations in GS strength. Ezer^18^ compared sea level difference between Atlantic City and Bermuda from tide gauges and altimeter with the total transport across the entire Oleander section, and found good agreement among different data. This suggests that the discrepancies in GS strength from pervious studies^15,17^ are likely due to different definitions and spatial variations. Indeed, linear trends in SSH and SSH-derived eddy kinetic energy (EKE) during 1993–2016 vary spatially, not only in magnitude, but also in the sign of the trends (Fig. 1a,c), suggesting that the GS changes are spatially-varying. West of 70°W in the GS region, SSH experiences positive trends exceeding 10 cm/decade, which can be partly explained by a northward shift of the GS, as shown in Fig. 1e. The positive trends in EKE on both sides of the GS in the same region (Fig. 1c) suggest that this northward shift is accompanied by a strengthening and/or a widening of the GS. To the east, SSH shows positive trends to the north of the GS and negative trends to the south, possibly corresponding to a weakening of the GS (Fig. 1f). This potential weakening of the GS is consistent with the decrease in EKE in the region (Fig. 1c). The trends in SSH for the period 1993–2011, corresponding to the study period of Ezer *et al*.^15^, reverse sign within the GS west of 70°W, an indication that the trends might also be sensitive to the time period. The trends in EKE during 1993–2011 have the same sign as those for the 1993–2016 time period, but are more pronounced.

To better understand the relationship of the GS properties and their variabilities in space and time, we used satellite altimetry data to determine and analyze the position, speed, width, and cross-front SSH difference of the GS over the region 80°W–50°W. The rest of the paper is organized as follows: First, we describe the data and methodology used to determine the GS properties. Second, we evaluate the properties derived from satellite altimetry by comparing them to those derived from *in situ* measurements, and demonstrate changes in the GS properties on interannual to longer time scales and their spatial variations. Third, we explore the correspondence between GS property changes and changes in sea level. Finally, we discuss the potential link between the GS and sea level rise along the U.S. East Coast.

## Methodology for Determining Gulf Stream Properties

The daily delayed-time, all-satellite-merged absolute dynamic topography (hereafter referred to as sea surface height, SSH) and absolute geostrophic velocity fields for our 24-year study period (1993–2016) on a 1/4° × 1/4° grid^19^ were used to determine GS properties, including position and width, maximum surface speed at the center, and cross-front height difference. While data quality improved by merging all available satellite observations, the sampling was inhomogeneous over time, which could have introduced errors due to low frequency variability. To investigate the impact of this sampling on our study, we used the two-satellite-merged products with homogenous temporal sampling to determine GS properties. The interannual variations and trends in the GS properties from these two products were similar, suggesting the impact of inhomogeneous temporal sampling was minimal, at least for this 24-year period.

**Figure 2:**
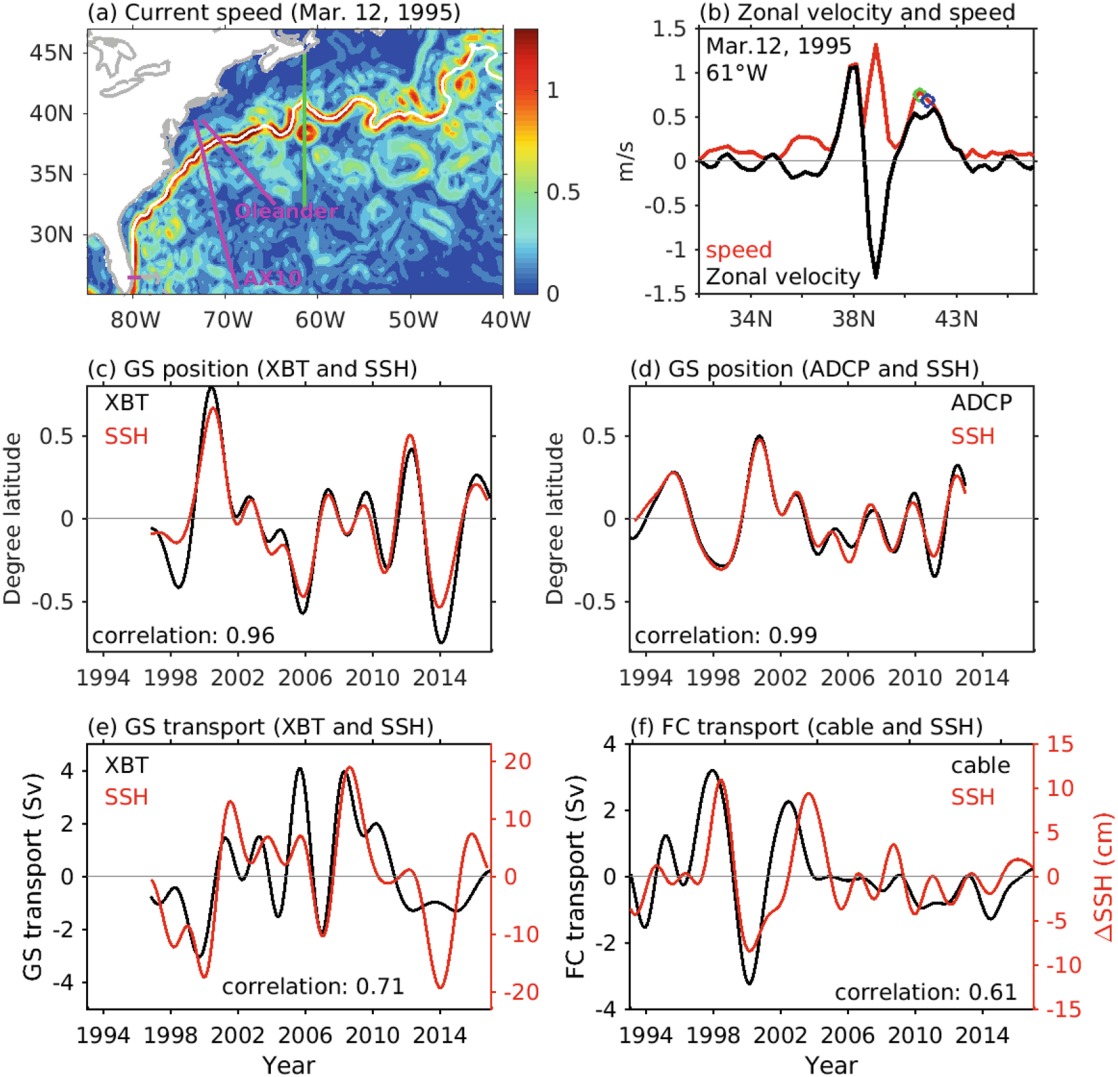
(**a**) An example of current speed in the GS region from altimeter data. Magenta lines denote the location of the Oleander line, AX10 transect, and the Florida Straits. (**b**) Zonal velocity (black line) and speed (red line) along 61°W (green line in (**a**)). Blue and green circles indicate the GS center determined from a SSH contour (first guess) and the maximum speed close to the SSH contour, respectively. (**c**) GS position at 72°W from the AX10 transect (black line) and SSH (red line). (**d**) GS position at 70°W from an ADCP (black line) and SSH (red line). (**e**) GS transport (0–800 m) derived from the AX10 transect (black line) and cross-front SSH difference (red line). (**f**) Florida Current transport from cable measurements (black line) and SSH differences across the Florida Straits (red line).

The center or core of the GS is generally determined as the location where its speed reaches a maximum. However, eddy activity in the GS east of 70°W makes it difficult to directly determine GS position from its maximum speed, since eddies may exhibit a larger current speed than the GS itself (Fig. 2a,b). Thus, the GS location may not be correctly defined using its maximum speed alone. In this study, we determined the center of the GS by using two criteria: the local surface current speed maximum (criterion one) in proximity to a specific SSH contour (criterion two). Details about this methodology, including how the SSH value for the contour was chosen, are described below.

In the methodology used here, the center of the GS at each grid point is positioned at the latitude of maximum surface current speed between 75°W–70°W, a region where the GS tends to follow a relatively straight path. The averaged SSH value at the GS center in this region, *h*_*gs*_, is used as the value of the SSH specific contour to estimate the position of the GS center. We then derive a first estimate of the GS position for the entire region (80°W–50°W) by following the contour of *h*_*gs*_, i.e., the center of the GS approximated at the latitude of the continuous SSH contour. Finally, we adjust this initial estimate for the GS center position by selecting the maximum velocity closest to the initial estimate along the line perpendicular to the local GS path. For statistical analysis purposes, only one GS position is chosen at each longitude grid for each daily field. In cases where the GS traveled through the same longitude multiple times, we chose the first latitude as the GS traveled downstream. This process was repeated for each daily SSH altimetry field.

Previous studies^20,21^ found that the cross-front SSH difference, ∆H, is proportional to the GS transport. In this study, we revisited this concept by comparing ∆H against two transport estimates using data from a high-density expendable bathythermograph (XBT) transect, referred to as AX10^22^, and a submarine cable across the Florida Straits^23,24^. Information about the Florida Current transport is publically available and has been described in Meinen *et al*.^24^. To determine ∆H across the GS, we selected the northern and southern boundaries of the GS at the location where the velocity changes sign. The difference of SSH at the southern and northern boundaries is defined as the cross-front ∆H, and the distance between the two boundaries defines the width of the GS.

The AX10 transect (Fig. 2a) between New York City and Puerto Rico crosses the GS at approximately 72.5°W; it has been continuously sampled since 1996, with approximately four transects per year. XBTs, which measure the upper ocean temperature to a depth of 850 m, are deployed approximately 10 km apart within the GS region. Salinity is determined based on historical temperature/salinity (T/S) relationships for each temperature profile^25^. Geostrophic velocity is computed using these T/S profiles and a reference depth of 800 m. The GS center position and transport are then determined following the same methodology as for the altimeter data. Salinity derived from temperature is valuable in estimating the strength of ocean currents^26,27^ in cases where only temperature is measured. However, the T-S relationship may break down as results of freshwater inputs from rivers and from atmosphere, which may partly contribute to the discrepancy in the GS properties determined from SSH and XBTs.

In addition, we used the hourly sea level data from tide gauges located along the U.S. East Coast from Florida to Maine to explore the relationship of coastal sea level with changes in the GS. The tide gauge data were obtained from NOAA’s Center for Operational Oceanographic Products and Services (https://opendap.co-ops.nos.noaa.gov/erddap/index.html) for the time period between 1985–2016, and all 43 tide stations were listed in detail in Domingues *et al*.^28^.

## Results

We evaluated the methodology for determining GS properties from altimeter SSH fields by interpolating the SSH-derived properties into *in situ* measurement time and location and comparing them with those from *in situ* measurements. As our analyses focused on changes in the GS on interannual to longer time scales, all current properties (location, speed, width, and cross-front ∆H) were smoothed by a Butterworth filter to remove signals with periods less than one year. We note that the significance of the correlations between the GS properties from different data and between sea level and the GS properties was determined based on the decorrelation time scale of one year.

We compared the GS location estimates with those from XBTs along the AX10 transect (Fig. 2c) and with current meter measurements along the AX32 transect also known as the Oleander line (Fig. 2d). We also compared the cross-front ∆H with transports from the AX10 transect (Fig. 2e) and with Florida Current transports from the submarine cable voltage measurements (Fig. 2f). The GS positions from altimeter data correlated well with data from the AX10 transects crossing 72.5°W and the Oleander section crossing 70°W, with correlations of 0.96 and 0.99, respectively. These correlations are well above the 95% significance levels of 0.46 and 0.44. On the other hand, the correlations of the cross-front ∆H with GS transports estimated from *in situ* measurements were not as high as those for the GS positions, with correlations of 0.71 and 0.61, respectively, for the AX10 transect and Florida Current transport across the Florida Straits. However, these values still exceed the 95% significance levels of 0.46 and 0.43. Consistent with previous studies, this suggests that changes in the cross-front ∆H are proportional to GS transport changes. Note that since our calculations did not extend to the Florida Straits, we used the SSH difference between the eastern and western boundaries of the Florida Straits along 26.5°N.

The GS properties derived from satellite altimetry and *in situ* measurements also agreed well in terms of long-term trends. As shown in Fig. 2, we found no trends for the GS position and transport from both SSH and *in situ* measurements along the AX10 and AX32 sections, as well as across the Florida Straits. These comparisons suggest that the satellite altimeter measurements reflect the GS changes observed by *in situ* measurements on interannual to longer time scales.

**Figure 3:**
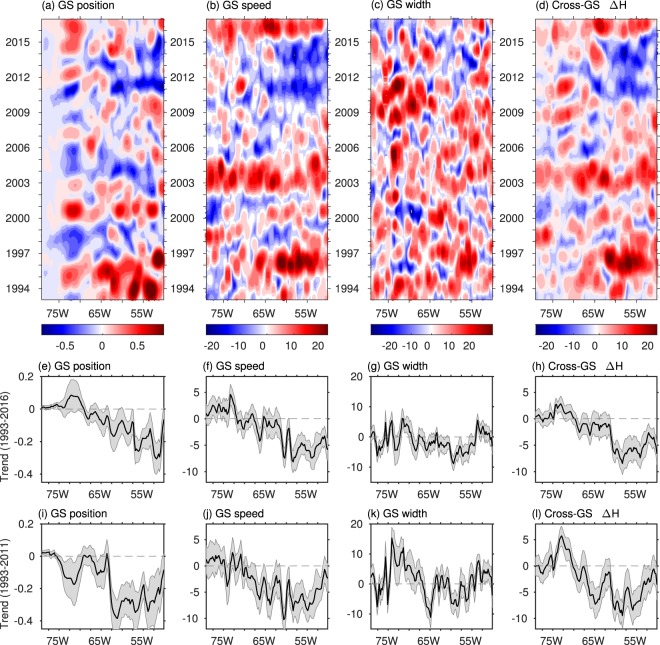
Anomalies of the GS position (**a**), speed (**b**), width (**c**), and cross-front SSH difference (**d**). Anomalies are computed by removing the annual cycle and performing a low-pass filter. (**e**–**h**) Linear trends during 1993–2016 at each longitude grid for the GS position, speed, width, and cross-front SSH difference, respectively. Gray shading indicates the 95% significance interval. (**i**–**l**) are similar to (**e**–**h**) but for the period 1993–2011. Units are degree latitude for GS position, cm/s for GS speed, km for GS width, and cm for cross-front SSH difference.

To place these results into spatial and temporal context, Fig. 3 shows the longitude-time diagrams of the GS properties and their trends at each longitude grid point. There is spatial variation in the magnitude and sign of the trends, and the trends are sensitive to our 24-year study period (1993–2016). Except for GS width, which does not show statistically significant trends on 95% significance level throughout the region, the trends for GS position, speed, and cross-front ∆H are dominated by values east of ~65°W after the GS passes the New England Seamount chain. To the west, the trends are weaker and vary between positive and negative values. The zonally-averaged GS position over the study region (not shown) experiences a southward shift, with a linear trend of −0.08 ± 0.02 degree latitude/decade. This southward shift in the GS center is accompanied by decreasing trends in both the GS speed and cross-front ∆H. The GS speed decreases at a rate of −1.74 ± 0.22 cm s^−1^/decade, and ∆H decreases at a rate of −2.21 ± 0.16 cm/decade.

To compare these results with previous studies^15,17^, we also computed the trends during 1993–2011 and found them to be generally consistent with those derived for the entire study period (1993–2016) east of 65°W. However, differences were observed for the region west of 70°W. Although marginal, the trends reverse sign from positive to negative for the GS position. This regional reversal in trends was also observed in the GS speed between 70°W–75°W (Fig. 3f,j). In the same region (70°W–75°W), the GS width shows relatively larger positive trends during 1993–2011 (Fig. 3k) compared to the trends during 1993–2016 (Fig. 3g). This suggests that the trends are sensitive to the study period.

The GS properties also demonstrate interannual variations, which explains the sensitivity of the long-term trends to the selected period. On average, the GS was in a more poleward position during 1993–1996, 2000, and 2015–2016, and in a more equatorward position during 1997–1999, 2004–2006, and 2010–2014. The GS speed and cross-front ∆H showed coherent changes, particularly east of 65°W, with positive anomalies during 1995–1996, 2002–2004, and 2015–2016, and negative anomalies during 2010–2014. The decreasing trends in the GS position, speed, and cross-front ∆H are largely attributed to the negative anomalies during 2010–2014. Changes in the GS width show less spatial coherence and are noisy in both space and time.

## Discussion

Two recent studies on the role of the GS on sea level rise along the U.S. East Coast, i.e., Ezer *et al*.^15^ and Rossby *et al*.^17^, produced different results, which may be partly due to the different variables examined in each of the studies. The agreement among different data^18^ suggests that the use of data from different observing platforms is not a major contributor to the different results. Ezer *et al*.^15^ explored the spatially averaged SSH gradient across the GS during 1993–2011 over the region 75.5°W–70°W, which is equivalent to the GS speed. They argued that the continuous weakening of the GS since 2004 corresponds to a reduced SSH difference across the GS, which results in an increase in SSH to the north of the GS and a decrease in SSH to the south. Rossby *et al*.^17^ investigated a layer transport measured by an acoustic Doppler current profiler at a depth just below the surface Ekman layer (~50 m). They computed transport within the region where velocity was in the same direction as the maximum velocity but found no significant trends for their 20-year study period (1993–2012).

Differences in the GS speed and transport and spatial variations in the GS properties can also account for the different results obtained by Ezer *et al*.^15^ and Rossby *et al*.^17^. As discussed in Rossby *et al*.^17^, the net sea level change across the GS, ∆H, is roughly determined using the cross-front momentum balance equation of ${\rm{\Delta }}H=\frac{f}{g}\langle v\rangle L$, where *f* is the Coriolis parameter, $g$ is acceleration due to gravity, 〈*v*〉 is the average GS speed, and *L* is the GS width. Therefore, both speed and width can induce changes in the cross-front ∆H.

**Figure 4:**
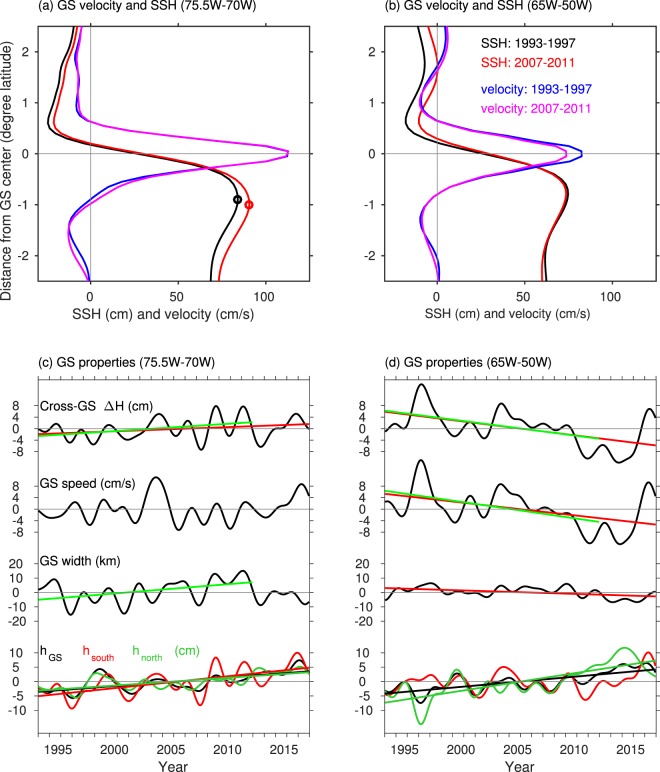
**(a)** SSH distribution across the GS averaged between 75°W–70°W during two 5-year periods: 1993–1997 (black line) and 2007–2011 (red line). Also shown is the corresponding velocity during the same periods: 1993–1997 (blue line) and 2007–2011 (magenta line). (**b**) Similar to (**a**) but for the region 65°W–50°W. (**c**) Time series of the cross-front SSH difference, the maximum speed and width of the GS, and the SSH at the GS center (black line) and those at its southern (red line) and northern (green line) boundaries averaged in the region 75°W–70°W. Linear trend for each property is computed for two periods: 1993–2016 (red line) and 1993–2011 (green line). (**d**) Similar to (**c**) but for the region 65°W–50°W.

To better demonstrate the changes in GS speed and cross-front SSH, we calculated the average velocity and SSH across the GS for the region 75.5°W–70°W over two 5-year periods: 1993–1997 and 2007–2011 (Fig. 4a). Note that the averages are calculated in a stream coordinate system, shifting with the GS meridional movement. Figure 4a shows that, compared to the first 5-year period (1993–1997), the SSH during 2007–2011 increased both to the north and south of the GS. The SSH increase to the south of the GS (6.22 cm) is more than twice larger than the increase to the north (2.80 cm), indicating an increase in the cross-front SSH difference. However, there are no significant changes in GS speed. The increase in ∆H during 2007–2011 is due to a widening of the GS. The control of the GS width on ∆H during 1993–2011 in terms of trend is clearly demonstrated by the time series of the GS properties shown in Fig. 4c. Consistent with the results shown in Fig. 4a, statistical analyses of the GS properties during 1993–2011 suggest increasing trends for both the cross-front ∆H (2.67 ± 1.29 cm/decade) and GS width (6.95 ± 2.78 km/decade), but no significant trends in GS speed. The SSH at the GS center and at both its northern and southern boundaries also show increasing trends, with a stronger trend at the southern boundary. For the entire study period (1993–2016), the trends in SSH are similar to those during 1993–2011. The cross-front ∆H shows a much weaker increasing trend (1.46 ± 1.02 cm/decade). However, no significant trends are found in the GS speed and width. The SSH increase on both sides of the GS cannot be explained by the proposed dynamic response of the SSH to a GS slowdown, which results in an increase in SSH to the north of the GS and a decrease in SSH to the south. During a relatively shorter time period, Fig. 4c is consistent with the findings of Ezer *et al*.^15^ of a steep decline in the GS speed west of 70°W between 2004–2011, when coastal sea level rise was accelerating, however it appears that this change was part of a decadal oscillation and not a long-term trend.

**Figure 5:**
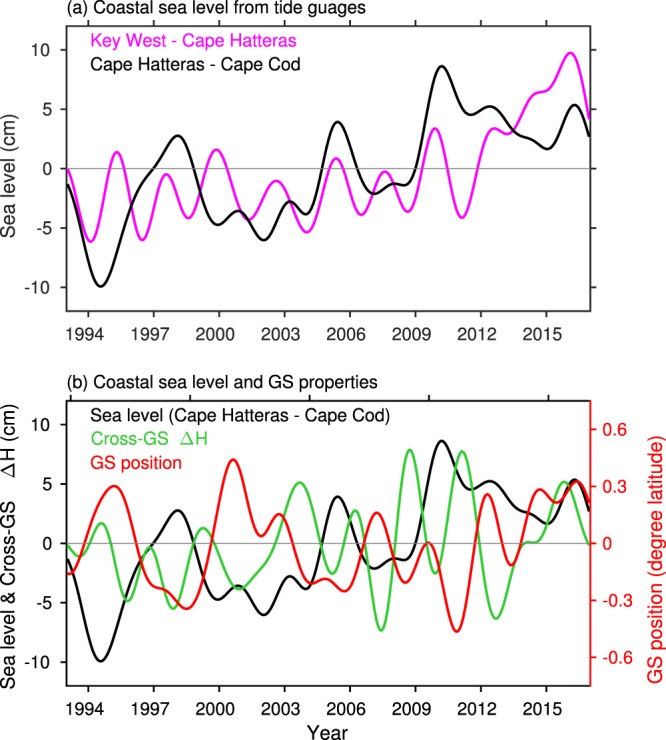
(**a**) Time series of sea level from tide gauges along the U.S. East Coast, averaged from 20 stations between Cape Hatteras and Cape Cod (black line), and that averaged from 15 stations between Key West and Cape Hatteras (magenta line). (**b**) Time series of sea level averaged between Cape Hatteras and Cape Cod (black line, same as in (**a**)) and the Gulf Stream properties, cross-front height difference (green line) and position (red line), averaged in the region 70°W–75.5°W. Units are degree latitude for GS position and cm for sea level and cross-front height difference.

To further evaluate the direct link of the coastal sea level changes with the GS, we analyzed the sea level from tide gauges along the U.S. East Coast. Consistent with previous studies^28,29^, we found differences in interannual variations of sea level north and south of Cape Hatteras. Figure 5 shows the averaged sea level between Cape Hatteras and Cape Cod (~70°W–75.5°W) as well as cross-front ∆H and the GS position averaged in the same region. The averaged GS velocity shows similar variations to the cross-front ∆H, therefore, it is not shown. The increasing trend in sea level (4.48 ± 1.74 cm/decade) is clearly demonstrated in Fig. 5. However, there is no corresponding decreasing trend in the GS properties to support a direct link between a slowdown of the GS and sea level rise. On interannual time scales, we also found lack of strong correspondence between sea level and the GS strength. After removing the linear trends, the correlations of sea level with the cross-front ∆H and GS velocity are −0.18 and −0.29, respectively, below 95% significance level of 0.40. The relatively high correlation (−0.58) between sea level and GS velocity in Ezer *et al*.^15^ was obtained for lower frequency (6–8 year period oscillations) signals during a shorter time period 1993–2011. Whereas the interannual variations shown in Fig. 5 are dominated by 2-3-year oscillations. Sea level and the GS position, however, show a significant correlation of −0.60 on interannual time scales. But this does not necessarily mean causation as both sea level and the GS position are linked to the North Atlantic Oscillation. The same conclusion holds when we examine the relationship of sea level changes in the region 70°W–75.5°W with the GS properties averaged over the entire study domain (50°W–80°W). This lack of direct link of coastal sea level changes with the GS variability is consistent with recent finding that the sea level rise pattern along the U.S East Coast is primarily determined by atmosphere-ocean interactions^28,29^.

For comparison, Fig. 4b shows the velocity and SSH distribution across the GS averaged over the region east of 65°W for the same two 5-year periods. Different from the region to the west, the eastern region shows a strong decrease in the cross-front SSH difference (9.07 cm), largely due to the increase in SSH to the north of the GS (7.55 cm). The width of the GS during the two periods is comparable, and the decrease in the cross-front ∆H is mainly due to the decrease in GS velocity, clearly demonstrated by the time series of the GS properties in Fig. 4d. Similar to the region between 70°W–75.5°W, the SSH at the center of the GS and at its northern boundary shows an increasing trend, but no significant trend is apparent for SSH at the southern boundary. The differences shown in Fig. 4 between the two regions suggest that the processes controlling GS changes on longer time scales may vary spatially.

**Figure 6:**
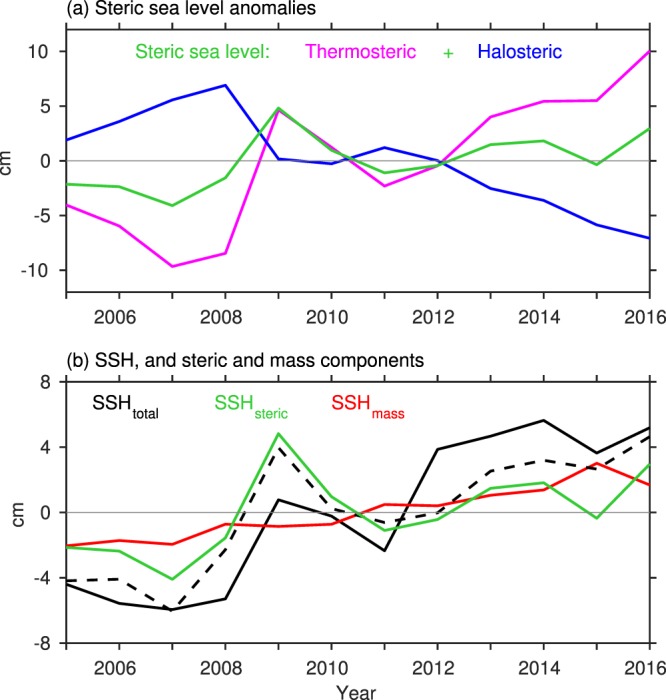
Yearly anomalies of (**a**) total steric sea level (green line), thermosteric (magenta line) and halosteric (blue line) sea level, and (**b**) altimeter SSH (black line), total steric sea level (green line), and mass-induced SSH (SSH_mass_, red line) averaged over the region 40°N–45°N and 50°W–65°W. The dashed black line in (**b**) is the sum of steric sea level and SSH_mass_. Unit is cm.

The two major contributors to regional SSH changes are variations in steric sea level due to thermal expansion and saline contraction, and mass-related sea level (SSH_mass_) changes. To investigate their contributions to the increase in SSH to the north of the GS, we examined the yearly steric sea level anomalies for the upper 2000 m layer from NOAA’s National Centers for Environmental Information available since 2005, and the SSH_mass_ using the Gravity Recovery and Climate Experiment (GRACE) data from the Jet Propulsion Laboratory (version 5, RL05M). Figure 6 shows the yearly anomalies of SSH, steric sea level, and SSH_mass_ averaged over the region 40°N–45°N and 50°W–65°W. The thermosteric sea level shows an increase of 14.08 cm from 2005 to 2016 (Fig. 6a). However, this increase is largely compensated by a decrease of −8.98 cm in halosteric sea level. The net steric sea level increase (5.10 cm) accounts for 53% of the increase in SSH (9.59 cm) during 2005–2016 (Fig. 6b). There is also an increase in SSH_mass_, of about 3.72 cm, explaining 38% of the SSH increase. This suggests that both ocean warming and increase in mass are important to account for the SSH increase to the north of the GS.

## Conclusions

Analyses presented in this study indicate that: (1) changes in GS properties are spatially dependent, i.e., a southward shift and weakening of the GS is found east of 65°W but not west of 70°W; and (2) sea level rise acceleration, as observed by tide gauges along the U.S. East Coast, is not directly linked to a slowdown of the GS.

During our 1993–2016 study period, the GS shows a southward shift east of 65°W after passing the New England Seamount chain. This southward shift is accompanied by a weakening of the GS, largely attributed to an increase in SSH to the north of the GS. Both ocean warming and increase in mass contribute to the positive SSH, with ocean warming playing a relatively larger role. West of 70°W, no statistically significant meridional shifts are found in the GS position, and the trends in the GS speed are weak. In the region east of 65°W, changes in the SSH north of the GS has relatively large control over the variations in the cross-front SSH difference. Whereas west of 70°W, the SSH changes that occur south of the GS dominate the variations in the cross-front SSH difference. These differences suggest that changes in the eastern and western regions of the GS may be controlled by different mechanisms. The GS properties also experience interannual variations, which are spatially dependent, particularly east and west of 65°W. Because of the strong interannual variations in GS properties, the linear trends are sensitive to the study period. Although the GS has experienced a slowdown on average since 1993, the speed up at the end of our study period suggests that this slowdown may be part of a decadal variation.

Our results suggest that the apparent contradiction of the impact of the GS on coastal sea levels from Ezer *et al*.^15^ and Rossby *et al*.^17^, i.e., a decrease in the average GS speed as opposed to a nearly constant GS transport, lies in the impact of the GS width. Consistent with Ezer *et al*.^15^, our examination indicates a marginal decrease in the GS velocity during 1993–2011. However, the cross-front SSH difference increases slightly due to a widening of the GS. Although our results do not support the idea that accelerated sea level rise along the U.S. East Coast is due to a slowdown of the GS, they do not eliminate the potential role of the GS-related processes on sea level rise acceleration. For example, a recent study^30^ demonstrated an increasing influence of GS warm core rings on the continental shelf south of New England, which can contribute to sea level rise through steric effect.

Direct steric effects should be minimal in shallow water where tide gauges are placed. However, steric sea level changes in the deeper ocean can impact coastal sea levels indirectly through dynamic processes as discussed in recent studies^31,32^. It is possible that the steric sea level changes in the deeper ocean induce variations in the pressure gradient which, in turn, result in mass exchange. Further investigation is needed to better understand the contributions of hydrodynamic and thermodynamic processes on SSH changes in the GS region, particularly in regions where the GS flows close to the coast, which could have critical societal implications under a global warming scenario. More detailed subsurface information from sustained *in situ* observations, combined with the more detailed spatial coverage from satellite measurements, will play a critical role in diagnosing the contributions of the different processes to sea level changes.
